# Supplementary material for: Semaphorin heterodimerization in cis regulates membrane targeting and neocortical wiring
Source: Nat Commun. 2024 Aug 16;15:7059. doi: 10.1038/s41467-024-51009-1 (PMC11329519; doi:10.1038/s41467-024-51009-1)
Supplement: Supplementary file 1 — Supplementary Information [file 41467_2024_51009_MOESM1_ESM.pdf]

Supplementary File

Semaphorin Heterodimerization in *cis* Regulates Membrane Targeting and Neocortical Wiring

Paraskevi Bessa[1, §], Andrew G. Newman[1, §], Kuo Yan[1], Theres Schaub[1], Rike Dannenberg[1], Denis Lajkó[1], Julia Eilenberger[2], Theresa Brunet[3,4], Kathrin Textoris-Taube[5,6], Emanuel Kemmler[7], Penghui Deng[1], Priyanka Banerjee[7], Ethiraj Ravindran[1], Robert Preissner[7], Marta Rosário[1, §], and Victor Tarabykin[1,9, §*]

Contents:

Supplementary Figures and Captions

Figure S1: Cell autonomous Satb2 deficits can be rescued by re-expressing Satb2.

Figure S2: Live imaging of *Satb2* mosaic deletion in organotypic cortical slices.

Figure S3: In situ hybridization screen against Satb2 dependent genes expressed in the cortical plate.

Figure S4: Semaphorin controls for in vivo experiments.

Figure S5: Alphafold2 predicted SEMA4D structures colored by prediction confidence.

Figure S6: Uncropped, unprocessed blots used in main figures.

Supplementary Note 1: Case Report SEMA4D LMU Dr. von Hauner Children Hospital

Supplementary Figures and Figure Captions

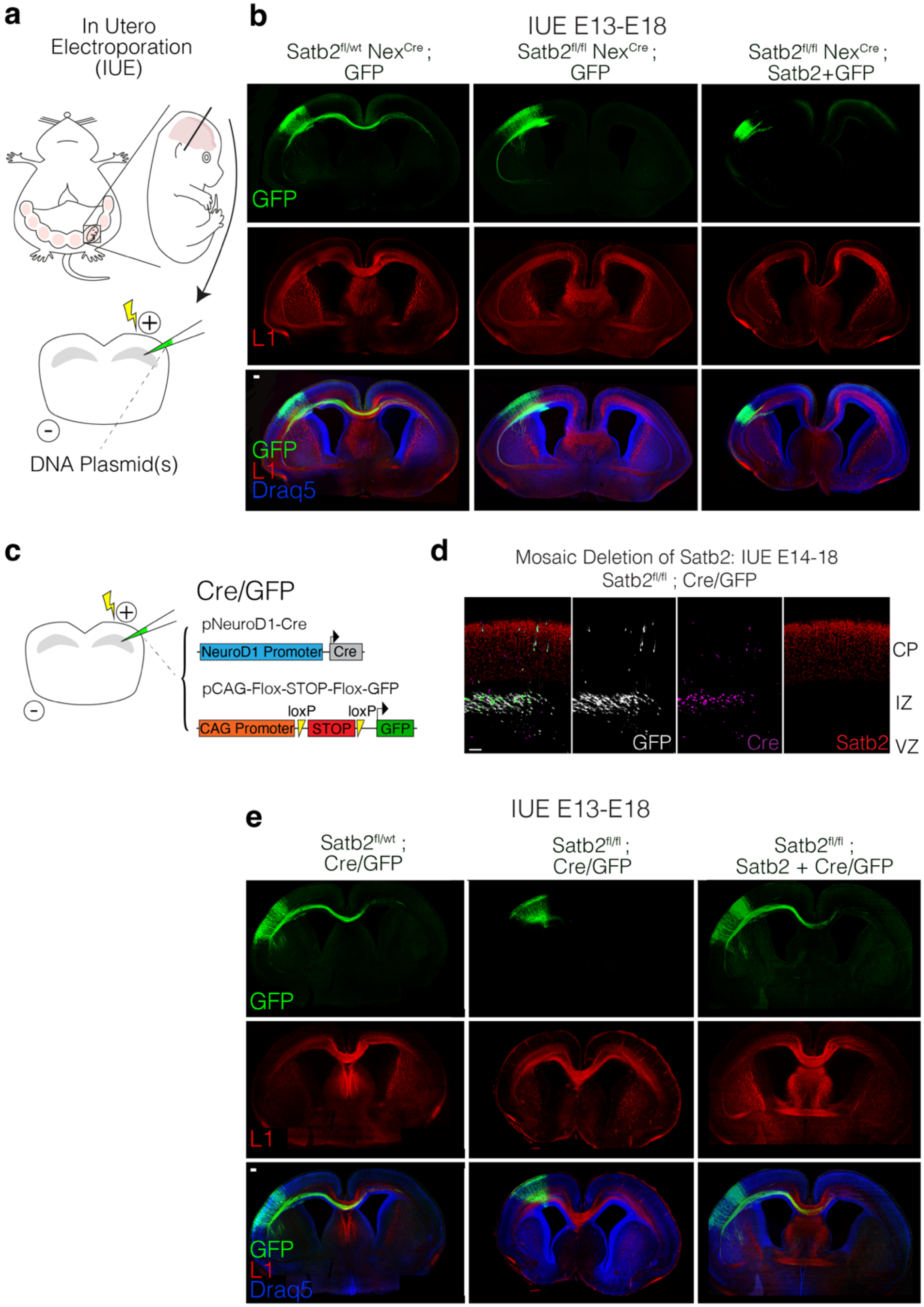

Figure S1 (previous page). Cell autonomous Satb2 deficits can be rescued by re-expressing Satb2. a Schematic showing In Utero Electroporation (IUE) technique involving the introduction of plasmid DNA into the lateral ventricle of live embryos using a capillary and an electric pulse. **b** Satb2 re-expression cannot rescue migration and axon extension in a completely Satb2-deficient cortex. (Left) IUE of GFP into a $Satb2^{fl/wt}$ Nex^{Cre} cortex results in normal migration and axon projection via the corpus callosum. IUE of GFP into a $Satb2^{fl/fl}$ Nex^{Cre} cortex results in cells that project via the internal capsule. Re-expression of Satb2 along with GFP into a $Satb2^{fl/fl}$ Nex^{Cre} cortex abolishes axon projections via the internal capsule and does not rescue axon projection to the midline (Right). L1 marks axonal tracts whereas Draq5 marks nuclei. Scale bar 100µm. **c** Strategy for mosaic deletion of Satb2 from newly born neurons by in utero electroporation. pNeuroD1-Cre confines Cre to post-mitotic neurons expressing NeuroD1. Upon Cre expression, floxed genomic Satb2 is excised, and the STOP cassette from pCAG-FSF-GFP is excised, resulting in GFP expression. **d** Cre/GFP plasmid strategy ensures all green cells observed are Cre positive and also Satb2 negative. These Cre positive, Satb2 negative cells that are labelled with GFP in the Ventricular zone (VZ) do not enter the cortical plate (CP) and remain in the Intermediate Zone (IZ). **e** Satb2 re-expression can rescue cells where Satb2 has been deleted in a mosaic fashion. Cre-injected neurons in *$Satb2^{fl/wt}$* (left) migrate and project axons normally. IUE of Cre into a *$Satb2^{fl/fl}$* cortex (middle) results in halted migration and an absence of axon projection. (Right): when *Satb2* is deleted by Cre and re-introduced into a *$Satb2^{fl/fl}$* cortex, migration into the cortical plate and axonal projections to the midline are restored. Scale bar 100 µm.

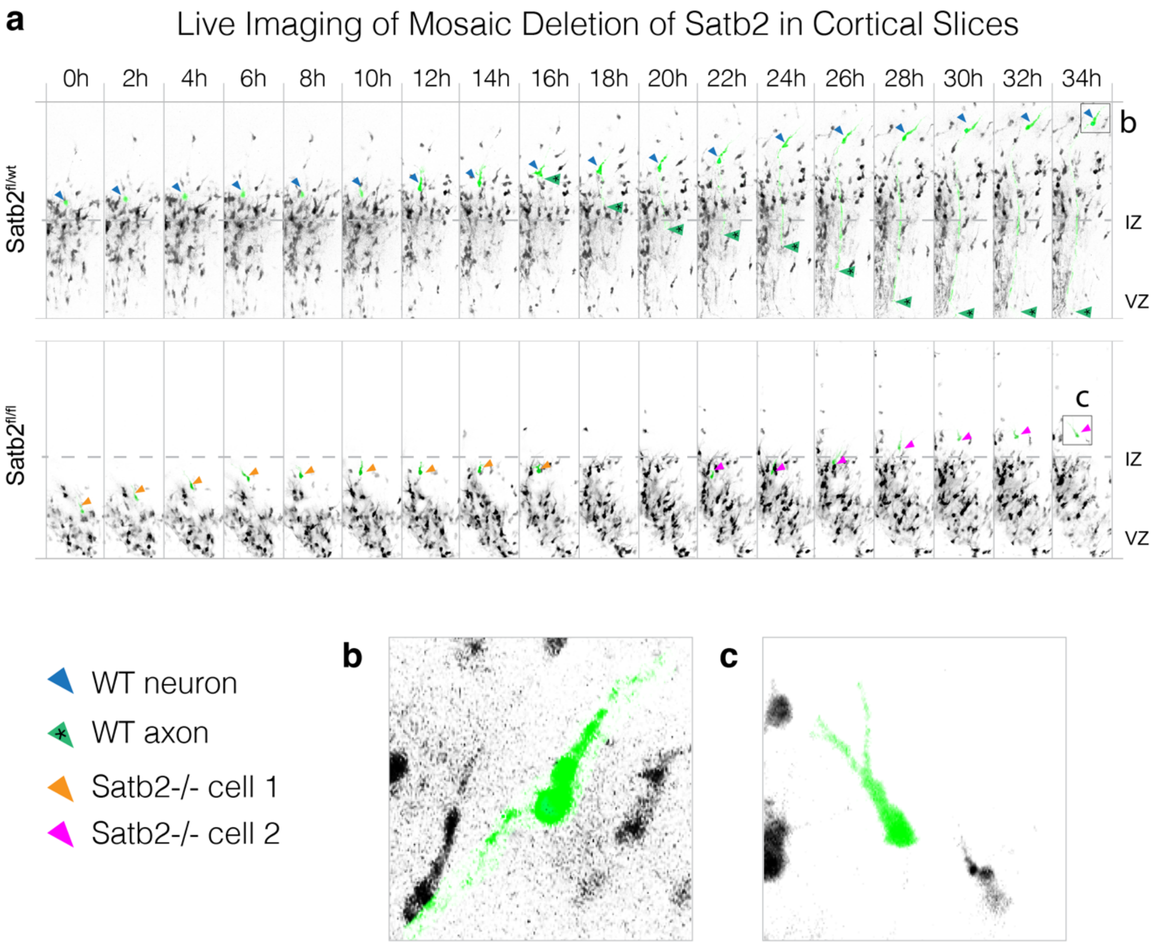

Figure S2. Live imaging of *Satb2* mosaic deletion in organotypic cortical slices. **a** pNeuroD1-Cre + pCAG-FSF-GFP *in utero* electroporation (IUE) visualized in live organotypic slice culture (250 μm thick). A *Satb2*$^{fl/wt}$ single cell (pseudocoloured green, yellow arrow) migrates towards the cortical plate and extends an axon (yellow arrow with asterisk) during the imaging time **b**. The bottom panel in **a** shows a *Satb2*$^{fl/fl}$ pseudocoloured cell (orange arrow) that fails to polarize and remains in the intermediate zone, and another *Satb2*$^{fl/fl}$ pseudocoloured cell (magenta arrow) that barely exits the IZ showing a bifurcated leading process **c**.

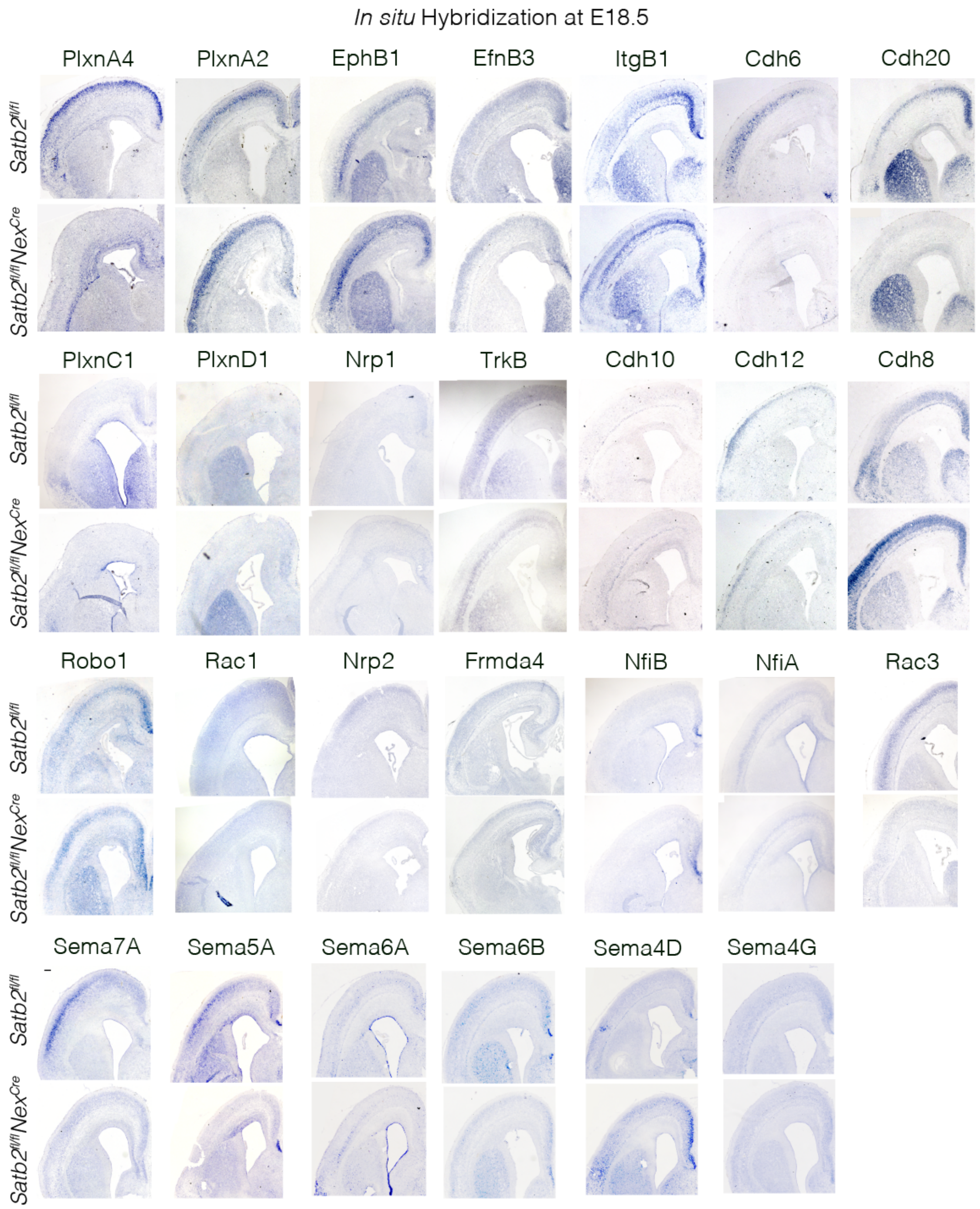

Figure S3. *In situ hybridization* screen against *Satb2* dependent genes expressed in the cortical plate.

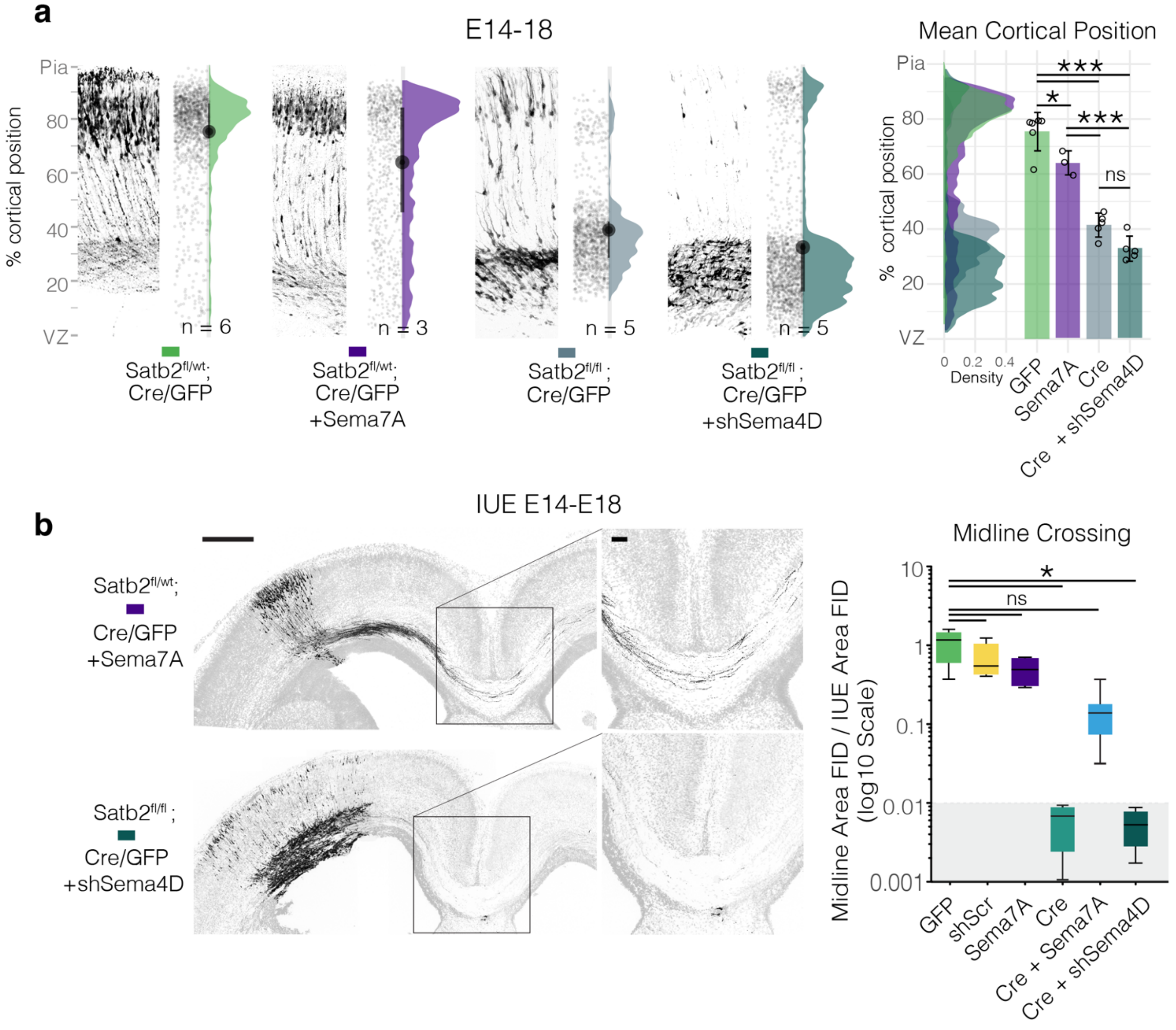

Figure S4. Related to Figure 1 & 5. Semaphorin controls for in vivo experiments. **a** Migration profiles of GFP, CRE (from Figure 1) alongside Sema7A overexpression in wildtype and Sema4D knock-down controls in Satb2-deficient cells. Migration profiles passed Shapiro-Wilk test for normality and were tested using one way ANOVA with Tukey's multiple comparisons. GFP vs Sema7A $p_{adjusted} = 0.0432$, GFP vs Cre $p_{adjusted} < 0.0001$, GFP vs Cre+shSema4D $p_{adjusted} < 0.0001$, Cre vs Sema7A $p_{adjusted} = 0.0002$, Cre vs Cre+shSema4D $p_{adjusted} = 0.1011$, Sema7A vs Cre+shSema4D $p_{adjusted} < 0.0001$. 'n' displayed on figure refers to one electroporated brain. **b** Left: midline crossing panoramas of Sema7A overexpression in wild-type $n_{brains}=4$ and Sema4D knockdown (shSema4D) in Satb2 mutant cells $n_{brains}=4$. Right: their midline quantifications in relation to GFP, shScr, Cre and Cre+ Sema7A conditions (data reproduced from Figures 1 & 5). Brown-Forsythe ANOVA with Dunnett's T3 multiple comparison test. GFP vs shScr $p_{adjusted} = 0.6412$, GFP vs Sema7A $p_{adjusted} = 0.1716$, GFP vs Cre $p_{adjusted} = 0.0289$, GFP vs Cre + Sema7A $p_{adjusted} = 0.052$, GFP vs Cre + shSema4D $p_{adjusted} = 0.0288$. Scalebar in panorama is 500µm, and 100µm in magnification. Midline n_{brains} GFP= 5, scScr = 5, Sema7A = 4, Cre = 4, Cre+Sema7A=8, Cre+shSema4D = 5.

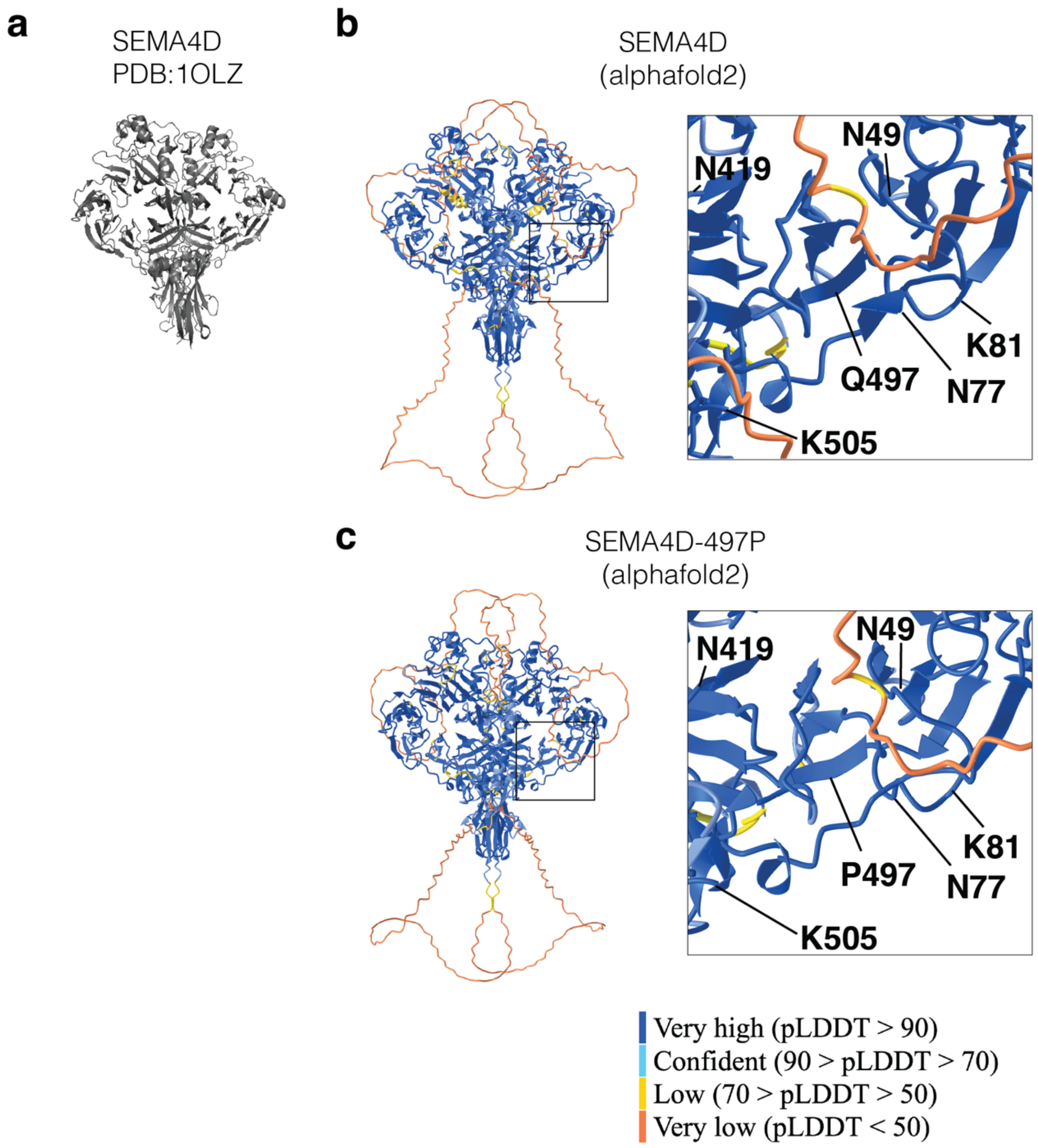

Figure S5. Related to Figure 6. Alphafold2 predicted SEMA4D structures colored by prediction confidence. a Solved crystal structure of human SEMA4D (PDB ID: 1OLZ). **b** Alphafold2 predicted structure of wild-type human SEMA4D. **c** Alphafold2 predicted structure of human SEMA4D with 497P mutation.

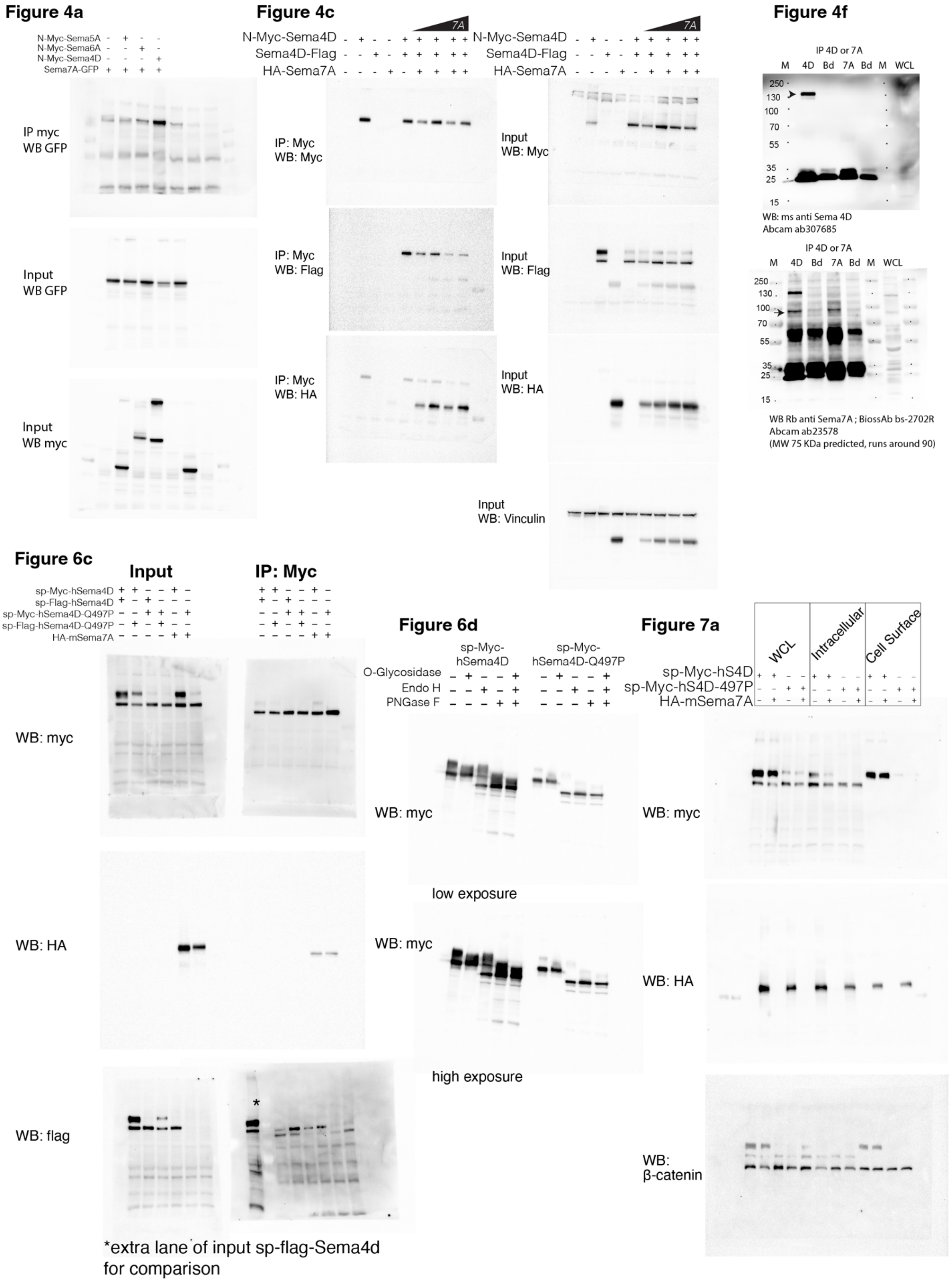

Figure S6. Related to the entire Manuscript. Uncropped, unprocessed blots used in main figures.

SUPPLEMENTAL NOTE 1

Case Report: SEMA4D LMU Dr. von Hauner Children Hospital

The patient was born at 40+2 gestational weeks after an uncomplicated pregnancy (weight 3390 g, APGAR 10/10). Delivery was by C-section due to obstructed labor. Newborn hearing and metabolic screening were normal. Due to a mild systolic heart murmur, echocardiography was performed showing a grade 1-2 tricuspid insufficiency. The further postnatal period was unremarkable. At the age of 5 months, the patient presented with a cluster of first generalized tonic-clonic seizures. Electroencephalography (EEG) showed left temporal spikes as well as intermittent slowing on the left parietal side. A brain MRI was performed showing only slightly hyperintense white matter that was interpreted as probably within normal range. Furthermore, the patient underwent several additional diagnostic procedures (lumbar puncture, laboratory and metabolism tests) that provided normal results. The family history was unremarkable except for the father, who had a simple febrile seizure when he was a baby.

The patient was seizure-free upon anti-seizure treatment with Levetiracetam and Oxcarbazepine. Seizures reoccurred with a monotherapy of Levetiracetam, so monotherapy was switched to Oxcarbazepine. Afterwards he did not show any seizures with Oxcarbazepine monotherapy until the age of 22 months and after that without any medication until the current age of 28 months. Following EEGs showed normal results. Psychomotor development was normal. The patient was walking at the age of 14 months and spoke short sentences at the age of 2 years. Clinical examination showed no dysmorphic features. Growth was regular, since weight, length, and OFC were 10 kg (17th centile), 80 cm (8th centile), and 51 cm (93th centile) respectively. Trio-based whole exome sequencing (WES) analysis detected the de novo missense variant (c.1490A>C, p.Gln497Pro) in SEMA4D (NM_006378.3).
