# Supplementary material for: Semaphorin heterodimerization in cis regulates membrane targeting and neocortical wiring
Source: Nat Commun. 2024 Aug 16;15:7059. doi: 10.1038/s41467-024-51009-1 (PMC11329519; doi:10.1038/s41467-024-51009-1)
Supplement: Supplementary file 3 — Description of Additional Supplementary Files [file 41467_2024_51009_MOESM3_ESM.pdf]

Title: Supplementary Data 1:
Description: Oligonucleotide sequences used for in situ probes and expression constructs

Title: Supplementary Data 2:
Description: Statistics performed including post hoc test type an p values of each quantification in this study

Title: Supplementary Data 3:
Description: Key Resources Table, a table of key reagents and resources used in the study
