# Supplementary material for: Semaphorin heterodimerization in cis regulates membrane targeting and neocortical wiring
Source: Nat Commun. 2024 Aug 16;15:7059. doi: 10.1038/s41467-024-51009-1 (PMC11329519; doi:10.1038/s41467-024-51009-1)
Supplement: Supplementary file 7 — Reporting Summary [file 41467_2024_51009_MOESM7_ESM.pdf]

nature portfolio

Corresponding author(s): Andrew Newman, Victor Tarabykin

Last updated by author(s): Jul 3, 2024

Reporting Summary

Nature Portfolio wishes to improve the reproducibility of the work that we publish. This form provides structure for consistency and transparency in reporting. For further information on Nature Portfolio policies, see our Editorial Policies and the Editorial Policy Checklist.

Statistics

For all statistical analyses, confirm that the following items are present in the figure legend, table legend, main text, or Methods section.

n/a	Confirmed	
☐	☒	The exact sample size (n) for each experimental group/condition, given as a discrete number and unit of measurement
☐	☒	A statement on whether measurements were taken from distinct samples or whether the same sample was measured repeatedly
☐	☒	The statistical test(s) used AND whether they are one- or two-sided<br>*Only common tests should be described solely by name; describe more complex techniques in the Methods section.*
☒	☐	A description of all covariates tested
☐	☒	A description of any assumptions or corrections, such as tests of normality and adjustment for multiple comparisons
☐	☒	A full description of the statistical parameters including central tendency (e.g. means) or other basic estimates (e.g. regression coefficient) AND variation (e.g. standard deviation) or associated estimates of uncertainty (e.g. confidence intervals)
☐	☒	For null hypothesis testing, the test statistic (e.g. F, t, r) with confidence intervals, effect sizes, degrees of freedom and P value noted<br>*Give P values as exact values whenever suitable.*
☒	☐	For Bayesian analysis, information on the choice of priors and Markov chain Monte Carlo settings
☒	☐	For hierarchical and complex designs, identification of the appropriate level for tests and full reporting of outcomes
☒	☐	Estimates of effect sizes (e.g. Cohen's d, Pearson's r), indicating how they were calculated

Our web collection on statistics for biologists contains articles on many of the points above.

Software and code

Policy information about availability of computer code

Data collection	No software was used for data collection
Data analysis	Data from electroporations was quantified using open imagej, and grouped and plotted using R. In some cases, tests for normality and statistical tests (ANOVA, Brown-Forsythe ANOVA, Kruskal-Wallis etc) were perfomed using GraphPad Prism 9.<br>Structural data was predicted using Alphafold2 and manipulated using PyMol.

For manuscripts utilizing custom algorithms or software that are central to the research but not yet described in published literature, software must be made available to editors and reviewers. We strongly encourage code deposition in a community repository (e.g. GitHub). See the Nature Portfolio guidelines for submitting code & software for further information.

Data

Policy information about availability of data

All manuscripts must include a data availability statement. This statement should provide the following information, where applicable:

- Accession codes, unique identifiers, or web links for publicly available datasets
- A description of any restrictions on data availability
- For clinical datasets or third party data, please ensure that the statement adheres to our policy

Source data are provided with this paper. Published datasets analysed are accessible from the Gene Expression Omnibus (GEO) database, www.ncbi.nlm.nih.gov/geo . Satb2-V5 ChIPseq accession GSE77005 [https://www.ncbi.nlm.nih.gov/geo/query/acc.cgi?acc=GSE77005], and Satb2KO RNAseq accession GSE68912 [https://

www.ncbi.nlm.nih.gov/geo/query/acc.cgi?acc=GSE68912].

Research involving human participants, their data, or biological material

Policy information about studies with human participants or human data. See also policy information about sex, gender (identity/presentation), and sexual orientation and race, ethnicity and racism.

Reporting on sex and gender	The case report pertaining to the patient holding the 497P mutation is devoid of indirect identifiers such as sex
Reporting on race, ethnicity, or other socially relevant groupings	The case report pertaining to the patient holding the 497P mutation is devoid of indirect identifiers such as ethnicity and ancestry.
Population characteristics	*Describe the covariate-relevant population characteristics of the human research participants (e.g. age, genotypic information, past and current diagnosis and treatment categories). If you filled out the behavioural & social sciences study design questions and have nothing to add here, write "See above."*
Recruitment	*Describe how participants were recruited. Outline any potential self-selection bias or other biases that may be present and how these are likely to impact results.*
Ethics oversight	*Identify the organization(s) that approved the study protocol.*

Note that full information on the approval of the study protocol must also be provided in the manuscript.

Field-specific reporting

Please select the one below that is the best fit for your research. If you are not sure, read the appropriate sections before making your selection.

☒ Life sciences ☐ Behavioural & social sciences ☐ Ecological, evolutionary & environmental sciences

For a reference copy of the document with all sections, see nature.com/documents/nr-reporting-summary-flat.pdf

Life sciences study design

All studies must disclose on these points even when the disclosure is negative.

Sample size	For in vivo experiments we observe pretty strong effect sizes in terms of neuronal behaviour in the form of cell migration and axon extension, where we have hundreds of neurons as observations across a minimum of 3 but typically more multiple biological replicates. With in vivo experiments there is always a trade-off of power vs what is the correct ethically appropriate number of animals to use.
Data exclusions	Occasionally embryonic brains from in vivo experiments were excluded due to adjacent uterine reabsorbtion or electroporation (capillary) artifacts.
Replication	Control and Experimental groups were always included within litter, and replicates across multiple repeats, controlling any covariates related to batch or minor variations in developmental timepoint.
Randomization	Experimental groups were assigned randomly at the time of experimental procedure or tissue collection.
Blinding	Quantification was performed single-blind, with cortex number used as the identifier.

Reporting for specific materials, systems and methods

We require information from authors about some types of materials, experimental systems and methods used in many studies. Here, indicate whether each material, system or method listed is relevant to your study. If you are not sure if a list item applies to your research, read the appropriate section before selecting a response.

Materials & experimental systems

n/a	Involved in the study	
☐	☒	Antibodies
☐	☒	Eukaryotic cell lines
☒	☐	Palaeontology and archaeology
☐	☒	Animals and other organisms
☒	☐	Clinical data
☒	☐	Dual use research of concern
☒	☐	Plants

Methods

n/a	Involved in the study	
☒	☐	ChIP-seq
☒	☐	Flow cytometry
☒	☐	MRI-based neuroimaging

Antibodies

Antibodies used	ms anti-Sema 4D/CD100, Abcam ab307685<br>Rb anti Sema 7A ; BiossAb bs-2702R Abcam ab23578<br><br>Rabbit anti-Satb2, Tarabykin Laboratory<br>Goat anti-GFP, Rockland<br>Chicken anti-GFP, Abcam<br>Mouse anti-Myc (9B11), Cell Signalling<br>Rabbit anti-Myc (71D10), Cell Signalling<br>Rabbit anti-HA (C29F4), Cell Signalling<br>Mouse anti-Flag, Cell Signalling<br>Rabbit anti-Cdk5rap2, Kaindl Laboratory<br>Rabbit anti-vinculin, Cell Signalling<br>Rabbit anti-Beta Catenin (D10A8), Cell Signalling<br>Mouse anti-GM130, BD Biosciences<br>Mouse anti-Cre Recombinase (clone 2D8), Millipore<br>Chicken anti-MAP2, Novus<br>Mouse anti-TAU-1 (clone PC1C6), Millipore
Validation	Rabbit Anti-Satb2 has been previously validated on our Satb2 knockout tissue.

Eukaryotic cell lines

Policy information about cell lines and Sex and Gender in Research

Cell line source(s)	HEK293T, Leibniz Institute DSMZ-German Collection of Microorganisms and Cell Cultures<br>Hela, Leibniz Institute DSMZ-German Collection of Microorganisms and Cell Cultures
Authentication	*Describe the authentication procedures for each cell line used OR declare that none of the cell lines used were authenticated.*
Mycoplasma contamination	Cells were regularly checked for mycoplasma contamination
Commonly misidentified lines<br>(See ICLAC register)	*Name any commonly misidentified cell lines used in the study and provide a rationale for their use.*

Animals and other research organisms

Policy information about studies involving animals; ARRIVE guidelines recommended for reporting animal research, and Sex and Gender in Research

Laboratory animals	Mouse (Mus Musculus): Satb2fl/fl (SATB2F) B6.-Satb2fl/fl Grosschedl Laboratory MGI:6363495<br>Mouse: (Mus Musculus): NexCre (NEXCRE) B6.-Neurod6tm1(cre)Kan Markus Schwab MGI:2668659
Wild animals	*Provide details on animals observed in or captured in the field; report species and age where possible. Describe how animals were caught and transported and what happened to captive animals after the study (if killed, explain why and describe method; if released, say where and when) OR state that the study did not involve wild animals.*
Reporting on sex	Embryos of both male and female sex were used. The phenotypes under investigation were not sex-dependent.
Field-collected samples	*For laboratory work with field-collected samples, describe all relevant parameters such as housing, maintenance, temperature, photoperiod and end-of-experiment protocol OR state that the study did not involve samples collected from the field.*
Ethics oversight	All mouse experiments were carried out in compliance with German law approved by the State Office for Health and Social Affairs, Council in Berlin, Landesamt für Gesundheit und Soziales (LaGeSo) under permissions G0079/11, G0206/16, G0184/20.

Note that full information on the approval of the study protocol must also be provided in the manuscript.

Plants

Seed stocks	*Report on the source of all seed stocks or other plant material used. If applicable, state the seed stock centre and catalogue number. If plant specimens were collected from the field, describe the collection location, date and sampling procedures.*
Novel plant genotypes	*Describe the methods by which all novel plant genotypes were produced. This includes those generated by transgenic approaches, gene editing, chemical/radiation-based mutagenesis and hybridization. For transgenic lines, describe the transformation method, the number of independent lines analyzed and the generation upon which experiments were performed. For gene-edited lines, describe the editor used, the endogenous sequence targeted for editing, the targeting guide RNA sequence (if applicable) and how the editor was applied.*
Authentication	*Describe any authentication procedures for each seed stock used or novel genotype generated. Describe any experiments used to assess the effect of a mutation and, where applicable, how potential secondary effects (e.g. second site T-DNA insertions, mosiacism, off-target gene editing) were examined.*
